# Challenges of Diphtheria Toxin Detection

**DOI:** 10.3390/toxins16060245

**Published:** 2024-05-26

**Authors:** Marta Prygiel, Ewa Mosiej, Maciej Polak, Katarzyna Krysztopa-Grzybowska, Karol Wdowiak, Kamila Formińska, Aleksandra A. Zasada

**Affiliations:** Department of Sera and Vaccines Evaluation, National Institute of Public Health NIH—National Research Institute, Chocimska 24, 00-791 Warsaw, Poland; mprygiel@pzh.gov.pl (M.P.); emosiej@pzh.gov.pl (E.M.); mpolak@pzh.gov.pl (M.P.); kkrysztopa@pzh.gov.pl (K.K.-G.); kwdowiak@pzh.gov.pl (K.W.); kforminska@pzh.gov.pl (K.F.)

**Keywords:** *Corynebacterium*, diphtheria toxin, methods of detection, in vivo methods, tissue culture cytotoxicity assays, Elek test, immunoassays, PCR, LAMP, biosensors

## Abstract

Diphtheria toxin (DT) is the main virulence factor of *Corynebacterium diphtheriae, C. ulcerans* and *C. pseudotuberculosis.* Moreover, new *Corynebacterium* species with the potential to produce diphtheria toxin have also been described. Therefore, the detection of the toxin is the most important test in the microbiological diagnosis of diphtheria and other corynebacteria infections. Since the first demonstration in 1888 that DT is a major virulence factor of *C. diphtheriae*, responsible for the systemic manifestation of the disease, various methods for DT detection have been developed, but the diagnostic usefulness of most of them has not been confirmed on a sufficiently large group of samples. Despite substantial progress in the science and diagnostics of infectious diseases, the Elek test is still the basic recommended diagnostic test for DT detection. The challenge here is the poor availability of an antitoxin and declining experience even in reference laboratories due to the low prevalence of diphtheria in developed countries. However, recent and very promising assays have been developed with the potential for use as rapid point-of-care testing (POCT), such as ICS and LFIA for toxin detection, LAMP for *tox* gene detection, and biosensors for both.

## 1. Introduction

Diphtheria is a serious infectious disease caused by *Corynebacterium diphtheriae*, *C. ulcerans* and *C. pseudotuberculosis*, although *C. diphtheriae* is indicated as the most common factor [1]. These Gram-positive bacteria most often colonise the mucous membranes of the throat and nose, sometimes also the larynx and trachea, less frequently the stomach, lungs, conjunctiva or mucous membranes of the genitals [2]. The bacteria may also infect skin and cutaneous lesions. Diphtheria toxin (DT) is the main virulence factor of *C. diphtheriae*, although toxigenic *C. ulcerans* and *C. pseudotuberculosis* strains have also been described. The toxin is extremely potent, with a minimal human lethal dose of less than 100 ng/kg of body weight [3]. The DT produced by the pathogens enters the bloodstream, spreads throughout the body and injures distant organs—mainly the heart, liver and central nervous system. The majority of deaths from diphtheria which result from severe complications of toxin absorption include acute systemic toxicity, myocarditis, cardiac failure, neurologic complications, primarily peripheral neuritis, and paralysis [4]. Recently, new *Corynebacterium* species with the potential to produce DT have been described, such as *C. silvaticum, C. rouxii* and *C. ramonii* [5,6]. DT is an approximately 58 kDa protein encoded by the *tox* gene of bacteriophage origin. *Tox* expression is regulated in response to changes in the Fe^2+^ level by the DtxR protein—a product of the DT repressor gene (*dtxR*) that presents on the bacterial chromosome [7,8]. There are three domains present in the structure of mature DT: catalytic, transmembrane, and receptor-binding. The N-terminal catalytic domain with ADP-ribosyltransferase activity is named fragment A, and the transmembrane and receptor-binding domains determine fragment B, which are linked to each other by a disulfide bond [9]. The lethal effect of the toxin begins with the interaction of the receptor-binding domain with a receptor on the eukaryotic cell surface, which induces endocytosis. Inside the endosome, the disulfide bond is reduced, and the low pH causes a change in the conformation of the DT, which increases its affinity for membrane lipid molecules. The transmembrane domain is then incorporated into the endosomal membrane, thus allowing for cytosolic exposure of the catalytic domain and initiation of the specific toxicity reaction. Translation of host proteins is inhibited by inactivation of elongation factor 2 (EF-2) due to its ADP-ribosylation, resulting in cell death [9,10].

**Figure 1:**
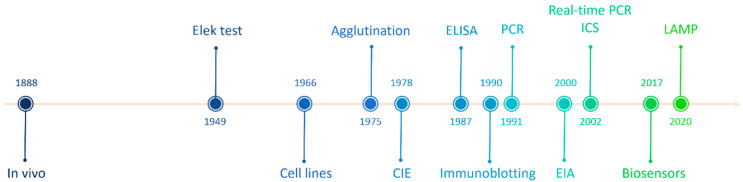
Diphtheria toxin detection in history.

Since 1888, when Pierre Emile Roux and Alexandre Yersin isolated the DT and demonstrated that systemic disease manifestations were related to the toxin presence, much research on DT detection has been conducted [11]. The detection of the toxin is the most important test in the microbiological diagnosis of diphtheria [12]. Below, we present various methods for DT detection described in the scientific literature (Figure 1).

## 2. Animal Models

The in vivo detection of DT is the traditional “gold standard”; however, currently, the test is used very rarely due to the availability of alternative methods. The detection of DT using in vivo methods is only possible if it retains the properties of a potent and lethal exotoxin. The first reports of the use of animals in diphtheria research date back to the second half of the nineteenth century, when Löffler reported the growth of diphtheria culture and postulated that the damage to the nasopharyngeal resulted from a soluble toxin. By 1888, Roux and Yersin soon confirmed the pathogenicity of *C. diphtheriae* for guinea pigs [13]. Animals injected with free filtrates of broth cultures of *C. diphtheriae* developed all manifestations of human diphtheria. This demonstrated that diphtheria exotoxin was the major virulence factor of *C. diphtheriae* [14]. The first studies using animals focused on understanding the mechanism of action of DT [13]. It was found that the sensitivity and resistance of different animal species to DT vary greatly. Man, dogs, rabbits, monkeys, and guinea pigs are susceptible species, while rats and mice are relatively resistant. Rats and mice can withstand 100 × the dose of the toxin that is a minimally lethal dose for guinea pigs. Interestingly, humans have been shown to be five times more sensitive to the deadly effects of toxin than guinea pigs [15]. DT is known to be lethal to susceptible animals and unvaccinated humans at very low doses of 100 ng/kg or even less [16]. The action of the toxin is known to inhibit protein synthesis, but its detailed biochemical involvement in the pathogenesis of the disease has not been fully known until recently [13]. It is also known that the toxin does not affect all tissues equally and some organs are more sensitive to DT than others. Cardiac failure and myocarditis are recognised complications of severe diphtheria in humans. Inhibition of protein synthesis in the guinea pig myocardium is similar to the clinical manifestations of the disease in man. Therefore, the guinea pig was selected as a model organism for DT toxicity testing [17]. Since then, the subcutaneous virulence test and the dermatonecrosis assay have been applied in guinea pigs to examine the toxigenicity of diphtheria isolates [18,19]. The presence of the toxin is recorded by the appearance of specific necrotic skin lesions (after 48 h) or the death of the animal (after 2 to 5 days). A rabbit skin test for investigation of *C. diphtheriae* virulence has also been proposed but is more often used for the assessment of diphtheria antitoxin [20].

In vivo tests are specific and sensitive but difficult to perform, of long duration, and with an inconvenient dependence on laboratory animal facilities. Before the introduction of in vitro cytotoxicity assays, animal assays were the only biological activity available from the DT detection method and are still considered the “gold standard” for toxigenicity tests [21]. It should be remembered that in vitro methods reflect only in vitro data, and it cannot be ruled out that isolates that are not toxic (e.g., non-toxigenic *tox* gene-bearing (NTTB) isolates) or weakly toxigenic in vitro are capable of producing DT during in vivo experiments [21]. The WHO Manual for the Laboratory Diagnosis of Diphtheria from 1994 mentioned the in vivo subcutaneous test for virulence in guinea pigs as the most reliable method for detection of DT production, but it did not recommend it for inexperienced laboratory staff. The current WHO laboratory manual for the diagnosis of diphtheria and other related infections, published in 2021 [22], does not mention in vivo toxin testing due to the availability of an alternative test (reducing animal suffering) such as the Elek test.

## 3. In Vitro Tissue Culture Cytotoxicity Assays

In vitro cytotoxicity assays are a suitable alternative to the in vivo virulence test. The first studies on DT using tissue cultures investigated the biochemical mode of action of the toxin [23]. These findings provided important data to justify the clinical symptoms that occur in humans. The information that DT inhibits protein synthesis was confirmed for the first time in vitro in the HeLa cervical cancer cell line by Strauss and Hendee [23]. Subsequent studies using guinea pig heart muscle tissue confirmed that biochemical heart injury was correlated with cardiac failure and tissue pathology observed in many cases of fatal diphtheria infections in humans [23]. Numerous studies have shown that cells from animals sensitive to DT are more sensitive to the toxin than cells from resistant animals; for example, guinea pig tracheal cells are 500 times more susceptible to the action of DT than murine fibroblastic cell line L929 [24,25]. Interestingly, resistance to the toxin has also been demonstrated in guinea pig embryos [24]. The inhibitory effect of DT on protein synthesis was also observed in tissue from skeletal muscle, liver, and brain. The results obtained with human cells and rabbit reticulocytes suggest that the reaction of inhibition of protein synthesis by DT is probably common to all sensitive mammalian cells [24]. Several tissue culture methods were developed to detect DT produced by live *C. diphtheriae.* Many research teams have tested a number of different cell lines for cytotoxicity of DT testing, among which the Vero line (African green monkey kidney cells) was probably the most popular [19,26,27]. Other cell lines used for the test primarily included rabbit kidney tissue culture cells, Chinese hamster ovary (CHO) cell line and HeLa cells [28,29,30]. The cytotoxic action of DT on cultured mammalian cells is based on the inhibition of protein synthesis, which leads to cell death [31]. In general, in most tissue culture methods for DT detection, *C. diphtheriae* culture filtrates or whole broth cultures are added to cell monolayers and incubated between 24 h to 5 days. A negative control (a toxin neutralised by an antitoxin) is used to exclude non-specific cytotoxic effects. Using dilutions of bacterial culture supernatants, cytotoxicity titres can be determined, which will enable quantitative determination of DT production by a given inoculum of the appropriate *C. diphtheriae* strain. Cytotoxic effects are usually directly visible or calorimetrically measurable. Moreover, Andre et al., in their study on monkey kidney tissue culture cells, confirmed that material with *C. diphtheriae* directly from throat swabs could be examined for cytotoxic activity [32].

The studies conducted by many research teams have confirmed that the results of optimised tissue culture cytotoxicity assays for DT detection demonstrate compatibility with the results of in vivo methods. The in vitro assays were confirmed to be specific, accurate and reliable in the detection and the quantification of biologically active DT produced by *C. diphtheriae* isolates. Moreover, the in vitro cytotoxicity assays are an order of magnitude more sensitive than the intradermal tests in vivo. The limits of DT detection in vitro are in the range between 10 and 100 pg/mL and are dependent on the method applied [19,27,29,33]. The in vitro tissue culture cytotoxicity assay is currently mainly used for measuring serum antitoxin levels. The use of the Vero cell line is recommended [22].

## 4. Immunological Assays

Immunoassay methods are based on the antigen–antibody reaction and are usually performed with mouse monoclonal antibodies (mAb) and, more rarely, equine polyclonal antibodies (pAb) directed against subunit A or B of DT. The limit of detection (LOD) for those methods is 0.1–4.0 ng/mL [34].

### 4.1. Elek and Modified Elek Tests

The immunodiffusion (or immunoprecipitation) assay commonly called Elek test was developed by Orjan Ouchterlony and Stephen D. Elek independently in 1948 [35,36]. The test is based on a precipitation phenomenon that occurs between the toxin and the antitoxin, creating visible precipitation lines on semi-solid media. The sensitivity of the test is comparable with that of the in vivo test on guinea pigs. In the test, toxin-positive, toxin-negative, and tested strains are spread on the agar surface in a straight line across the plate and at an angle to the paper strip soaked in the antitoxin and placed on the medium with bacteria inoculum. Following the reaction, precipitation lines are formed between the antitoxin strip and the line of bacterial growth, indicating toxin production [36,37]. The test results are visible after 24 to 48 h. The recommended basal medium is the improved agar base supplemented with serum. The medium might be supplemented with rabbit, calf, adult bovine serum and newborn bovine serum, with the best standard recommended by the WHO being newborn bovine serum [12,22]. The reliability of the test is strongly related to the quality of the medium and antitoxin used, and therefore each batch of medium, serum and antitoxin should be checked before application for the diagnostic test [12,22,37].

The first modification of the Elek test was proposed by Feldman et al. [38]. They used discs soaked with an antitoxin around which the culture of the strains was inoculated. This test was used in countries of the USSR during the diphtheria epidemic in the 1990s. [39]. Other modifications of the Elek test was proposed in the 90s by Engler et al. [40]. In their study, they paid attention to the volume of the medium, concentrations of the antitoxins, inoculum density and the distance of inoculation from antitoxin disks. By changing all these parameters, they were able to reduce the incubation time. A thinner medium with less density and a closer distance between the antitoxin disc and inoculum reduced the time to obtain the results of the test to within 16 to 24 h. Moreover, the authors suggested that extending the incubation time may result in the occurrence of non-specific lines of precipitation. Further modifications proposed in the scientific literature concerned the medium used for the test, for example, the application of Columbia blood agar base instead of Elek base medium [41] and the application of a modified medium for simultaneous Cystinase and Elek tests [42].

The test originally proposed by Elek replaced the in vivo virulence test in guinea pigs, the use fo which, due to high costs, the risk of accidental self-inoculation and extended waiting times, has been reduced in most laboratories [21]. Currently, the Elek test (and the modified Elek test) is recommended as the “gold standard” in diphtheria diagnostics. Both conventional and modified Elek tests are recommended in the current version of the WHO laboratory manual for the diagnosis of diphtheria and other related infections [22]. The manual presents a step-by-step procedure for performing the test, including important quality recommendations. However, it must be underscored that the tests require experienced staff to avoid misinterpretation of the results. The inconvenience of using the Elek method in routine diagnostics may be the problem of limited access to supplies of diphtheria antitoxin. Antitoxin is available from only a few sources worldwide, mostly from India [22]. It is therefore extremely important to look for alternative, more easily achieved methods.

### 4.2. Counterimmunoelectrophoresis

Counterimmunoelectrophoresis (CIE) was originally described by Bussard and Huer in 1959 and used for the detection of hepatitis virus antigens (HBsAg) [43]. Almost 20 years later, the method was proposed for the detection of DT in *C. diphtheriae* cultures [44]; this method refers to the formation of a precipitation line in agar. The process is a modification of immunoelectrophoresis and takes place under the influence of an electric field. In CIE, the antigen and antibody move in opposite directions, forming precipitates at the point of their connection. In the obtained results, the presence of a precipitation line indicates the corresponding antigen to the antibodies. In the CIE test, the DT production can be detected within 16 to 24 h. The test was developed using five toxigenic *C. diphtheriae* strains, one non-toxigenic *C. diphtheriae* strain, one *C. hemolyticum* and three unspeciated diphtheroid isolates, and the influence of the culture medium and culture conditions on the test results was indicated.

### 4.3. Agglutination Methods

The application of various agglutination-based tests has been proposed for DT detection, including latex agglutination, coagglutination, slide agglutination and haemagglutination. Holmes and Perlow developed a reverse passive haemagglutination (RPHA) test for the detection of DT in 1975 [45]. The RPHA test was conducted in microtiter plates with V-bottom wells. The purified antitoxic antibodies were coupled to sheep erythrocytes and agglutination occurred after overnight incubation at 4 °C when DT was present in *C. diphtheriae* culture supernatant. The detection limit of DT by the RPHA test was about 200 pg/mL. However, the study was conducted only on the reference strain *C. diphtheriae* PW8 and on the mutants of the strain C7 of *C. diphtheriae* and compared to an immunodiffusion method. Jalgaonkar and Saoji applied a coagglutination test, in which Cowan I strain of *Staphylococcus aureus* was used as a carrier particle to coat antibodies [46]. An antibody to the DT was coupled to protein A of *Staphylococcus aureus*. Protein A combines with the Fc portion of the antibody, enabling the antigenic Fab regions to be freely exposed on the surface of the *S. aureus* cells. The sensitised *S. aureus* cells were mixed with the bacterial culture supernatants on a slide and agglutinate within 10 min if the DT was present. The test results were consistent with the conventional guinea pig pathogenicity test, but it was verified only on eight *C. diphtheriae* clinical isolates, including five toxinogenic isolates. A reversed passive latex agglutination (RPLA) test for the detection of toxigenic *C. diphtheriae* isolates was developed in 1997. In this assay, the affinity-purified antibodies were attached to latex particles and reacted with the soluble DT [47]. For the RPLA assay, bacterial strains were grown in Elek broth medium with 10% calf foetal serum at 34 °C with shaking. It was demonstrated that 4 h of cultivation was not enough for DT production in slow toxin producer isolates, while 6 h of cultivation was enough to obtain a clearly positive result. In a U-bottom microtiter plate, the bacterial culture supernatants were serially diluted twofold. Subsequently, an equal volume of sensitised latex was added to each well. The microtiter plate was covered, shaken thoroughly and incubated at room temperature. Agglutination was assessed visually after 6 h. If DT was present in the tested supernatants, agglutination occurred as a result of the formation of a molecular lattice and a dispersed layer was formed at the bottom of the well. In the absence of DT, the latex particles sedimented to the bottom of the well and formed a tight button. The DT titre was determined as the reciprocal of the highest dilution in which agglutination was recorded. The RPLA assay enables the rapid detection of the DT in 12 h, of which 6 h are required for bacteria cultivation for toxin production, and an additional 6 h are needed to observe latex agglutination. The limit of detection of DT by the RPLA assay was about 5 ng/mL. The assay results were in agreement with the results of PCR and the Vero cell cytotoxicity assays. But the assay was evaluated only with seven *C. diphtheriae* isolates—fve positive and two negative for toxin production.

### 4.4. Enzyme-Linked Immunosorbent Assay

The enzyme-linked immunosorbent assay (ELISA) for DT determination was described in [48]. ELISA can be a qualitative or quantitative method, with quantitative immunoassay methods based on mAbs being easier to standardise [34]. The results of sandwich ELISA cover the results of other methods for DT detection, such as the Elek test or genetic identification of the *tox* gene, and are characterised by high sensitivity (approximately 99%) and specificity (100%). But occasional false positive results were also documented. The detection limit of DT is of less than 1 ng/mL [34]. The use of ELISA to determine the level of the toxin production by strains may be helpful in assessing the pathogenic potential of isolates [49]. This is very important because diphtheria epidemic isolates cause more severe forms of infection in unvaccinated children when they secrete greater amounts of the toxin [50]. There are many difficulties in the detection of a weakly toxigenic strain of *C. diphtheriae*. Some authors have suggested that ELISA may be useful as a reference in vitro method for detecting isolates with questionable toxin production. The research carried out by Simonova et al. showed that *Corynebacterium* strains with low levels of DT were not detected by the Elek test and gave positive results in ELISA [34]. Although the ELISA method is suitable for detecting toxigenic diphtheria isolates in routine practice, this is a labour-intensive and time-consuming assay. Another disadvantage is that there are no commercially available kits and all ELISA tests are based on the validation of the internal method.

### 4.5. Enzyme Immunoassay

The enzyme immunoassay (EIA) is a modification of the ELISA test for rapid diagnostics [18]. EIA is also characterised by a very high diagnostic sensitivity (LOD is 0.1 ng/mL). In a comparative study of 220 toxigenic *Corynebacterium* isolates, the results obtained by the EIA method correlate 100% with the results obtained by the Elek test [51]. Engler et al. also reported 100% consistency of the results of both methods [51]. This shows that it is a highly accurate and specific method for the detection of toxigenicity. EIA provided a result within 3 h of colony selection compared to 24 h for the modified Elek test and 48 h for the conventional Elek test. Furthermore, the interpretation of the EIA results was easier than for that of the Elek test. Isolates that showed weak precipitation lines in the Elek test gave a good visible colour change in the EIA [51]. To increase the detection of isolates with the biological activity of DT, the standardisation of the density of the inoculum and the incubation time in broth are very important, particularly among isolates producing low levels of the toxin [51].

### 4.6. Immunoblotting

Whereas the ELISA and EIA methods are good for testing culture supernatants, the immunoblotting method is better suited for studies of whole lysates of pathogenic corynebacteria, and this is only a qualitative method. Nitrocellulose membranes are used as solid phases that bind to protein more strongly than solid plastic phases (ELISA, EIA). The LOD (analytical sensitivity) of this method is about 5–10 ng/mL [52]. Immunoblot detection can be characterised by a low percentage of false positive results for nonspecific binding. To improve the specificity of the assay, proteins were/are denatured and separated by SDS-PAGE electrophoresis before detection. The results can be obtained after 11 h, but for weakly toxigenic isolates, the test must be extended to approximately 24 h [52]. The limitation of immunoblotting assays is the inability to distinguish between biologically active and inactive toxins. Despite many advantages (high specificity and sensitivity), due to time and labour consumption, immunoblotting is not recommended for routine diagnostic use [19,52,53].

### 4.7. Immunochromatographic Strip Tests

The reagents previously used in the enzyme immunoassay (EIA) [51] were applied to develop an immunochromatographic strip test (ICS). The ICS test uses equine polyclonal antitoxin coated onto nitrocellulose membrane as the capture antibody, and colloidal-gold-labelled monoclonal antibodies specific for fragment A of the DT molecule as the detection antibody. The assay enables rapid detection of DT from pure *C. diphtheriae* clinical isolates as well as from clinical specimens (throat swabs) [54]. Using the ICS test, a definitive toxicity result is available within 3.5 h for pure isolates. *C. diphtheriae* colonies from Columbia blood agar should be suspended in serum-supplemented Elek broth (SSEB) and incubated for 3 h at 37 °C. In the case of toxicity testing directly from throat swabs, 16 h of incubation in the SSEB medium is required. Subsequently, an ICS test strip should be added to each broth tube, and the result is available within 10 min. In addition, 100% consistency has been demonstrated between ICS and Elek test results for 915 pure clinical *Corynebacterium* isolates (851 *C. diphtheriae* and 64 *C. ulcerans*), and 99% agreement for results obtained for throat swabs directly inoculated into broth cultures (848 of 850 specimens), which results in a sensitivity and specificity for the ICS test of 98% and 99%, respectively. The limit of detection of the ICS test is 0.5 ng of DT per ml. In conclusion, the ICS test is reliable, highly sensitive and specific. The test is simple to perform and enables the rapid detection of DT. Moreover, this method does not require any specialised equipment and the results of ICS test are easy to interpret. Unfortunately, the production of the ICS strips ceased [55].

Another study performed by Melnikov et al. [56] presented lateral flow immunoassay (LFIA) as a method for detection of DT, in which a pair of monoclonal antibodies specific to subunit B of DT, previously used to develop the ELISA assay by Simonova et al. [34], was applied. In the case of the ICS test, the monoclonal antibody against subunit A of DT was used as the detecting antibody [54]. LFIA test strips were manufactured under the H2020-MSCA-IF-2018 (843405-DIFTERIA) research programme by Senova (Weimar, Germany). A pair of mouse monoclonal antibodies to the DT B subunit—IgG1anti-DT 675-3 (capture antibody on the solid support) and IgG1 anti-DT 676-3 labelled with colloidal gold (detection antibody) and polyclonal goat anti-mouse IgG (control antibody)—were applied in LFIA. The test strips were placed into lateral flow cassettes. LFIA for DT detection was validated with the use of 322 corynebacterial cultures as well as 360 simulated diphtheria specimens, which were obtained by adding test strains of corynebacteria to human throat samples. In addition, 24 h corynebacterial cultures on Columbia blood agar were inoculated on Elek broth, and after 6 h of incubation at 37 °C, 100 µL of the liquid culture was added to the LFIA cassette, with the result being available within 15 min. The simulated diphtheria specimens were plated on selective Hoyle’s tellurite agar and cultivated for 18–24 h at 37 °C. Next, a mixture of 5–10 grey/black colonies, characteristic of corynebacteria, were inoculated on Elek broth and then cultivated for 6 h at 37 °C. It was shown that LFIA detected DT in corynebacterial cultures with high accuracy. The LFIA results obtained for 321 of the 322 corynebacterial strains were consistent with the results of the Elek test. It was demonstrated that the diagnostic sensitivity of the LFIA for DT detection on bacterial cultures was 99.35%, and the specificity was 100% [56]. Compared to the Elek test, the LFIA test is much simpler and faster to perform. Implementation of the LFIA into routine usage may speed up the detection of toxigenic corynebacterial isolates.

## 5. Nucleic Acid Amplification Tests

Nucleic acid amplification tests (NAATs) are rapid molecular techniques that enable the detection of small amounts of genetic material in tested samples. NAATs for toxigenicity determination are based on the detection of the *tox* gene encoding DT [22]. Since the presence of the *tox* gene does not necessarily determine the production of DT—as non-toxigenic strains bearing the *tox* gene (NTTB) of *C. diphtheriae*, *C. ulcerans*, and *C. pseudotuberculosis* have been reported [19,57,58,59,60,61]—all positive NAATs results in patients suspected of diphtheria must be phenotypically confirmed, for example, by the Elek test [22]. However, *tox*-positive NAATs results can be considered for further preventive treatment of patients without waiting for the Elek test results [22,62]. In contrast, *tox*-negative results are considered definitive and do not require confirmation by other methods [22,62]. The use of NAATs allows for the quick exclusion of toxigenicity in tested isolates and prevents unnecessary further phenotypic measures for toxin presence. Moreover, unlike phenotypic tests, NAATs can detect NTTB isolates, which pose a potential epidemiological threat as they could theoretically reactivate *tox* expression after phage conversion or DNA recombination [22].

### 5.1. Polymerase Chain Reaction

The first NAATs used for *tox* detection were based on conventional polymerase chain reaction (PCR), in which fluorescently stained PCR products are resolved on an agarose gel, and were described in the 1990s [63,64,65,66]. Among the conventional PCR systems, those developed by Pallen et al. [64], Hauser et al. [65], and Nakao and Popovic [66] have likely been the most widely used [57]. The PCR system described by Pallen et al. [64] focuses on the amplification of fragment A of the *tox* gene (*toxA*), encoding the catalytic subunit of the toxin. In contrast, the system by Nakao and Popovic [66], an extension of the previous system with an additional primer set, also amplifies fragment B of the gene (*toxB*), which encodes the translocation and receptor-binding domains [63,66]. The PCR by Hauser et al. [65] targets both the A and B fragments in a single amplicon (*toxAB*) [65]. Most assays using these protocols have been performed on pure bacterial cultures, but direct *tox* detection from clinical specimens has also been reported [57,66,67]. The protocols can be used on preparations of simple boiled cells or pure extracted DNA [22]. The LOD calculated for the Nakao and Popovic [66] test ranges from 50 to 1500 CFU/PCR mixture [66,67]. The *toxA* amplifying primer set was found to be more sensitive than the *toxB* primer set [66,68]. Data from numerous studies have shown 100% correlation between the results of these three conventional PCR systems and phenotypic methods, such as in vivo ADP-ribosylation activity and the Elek test [19,57,64,65,66,67,69,70,71,72]. However, it should be noted that NTTB strains identified by PCR but not by phenotypic methods have been described [19,58,59]. The three most commonly used conventional PCR systems, although originally designed for *C. diphtheriae* toxicity testing, have frequently been used for the effective amplification and detection of the *C. ulcerans tox* gene. This occurred despite approximately 5% divergence between the *C. ulcerans* and *C. diphtheriae tox* gene nucleotide sequences [57,73]. Specific detection of the *C. ulcerans tox* gene alone—without the *C. diphtheriae tox* gene—was possible using primers developed by Sing et al. [57,73,74].

Faster and more sensitive detection of toxigenicity compared to conventional PCR was achieved using real-time PCR, a method that monitors the amplification of target DNA during the process. The first real-time PCR assay for toxin detection was developed at the Centers for Disease Control and Prevention (CDC) and published in 2002 [22,67]. This method involved the amplification of *toxA* and *toxB* fragments and their real-time detection using hydrolysis probes (TaqMan). The analytical sensitivity of the assay was estimated at 2 CFU (17 fg of genomic DNA), which was 750-fold higher than that of the conventional PCR method by Nakao and Popovic [66,67]. Higher sensitivity of the test compared to conventional PCR was also demonstrated in clinical specimens [57,67]. In the authors^’^ evaluation, the test achieved 100% sensitivity and specificity, although further studies showed that it was unreliable in assessing the toxicity of some *C. ulcerans* isolates, resulting in atypical or false-negative results [75,76]. These observations led Schuhegger et al. [74] to design primers as well as TaqMan hydrolysis probes targeting regions of the *tox* gene that are conserved in both *C. diphtheriae* and *C. ulcerans* [57,74]. This assay demonstrated 100% sensitivity and specificity with a detection limit of 100 fg of DNA per reaction, making it 25 times more sensitive than the conventional PCR designed by Hauser et al. [65,74]. This method has also been successfully used by others to identify the *tox* gene of both *C. diphtheriae* and *C. ulcerans* [57,77]. Real-time PCR for the *tox* gene was also designed in another popular real-time detection format using LightCycler hybridisation probes [57,71]. Evaluation showed that the method had 100% sensitivity and specificity with a detection limit of 100 fg per reaction. Moreover, LightCycler melting curve analysis allowed for the differentiation of *C. diphtheriae* and *C. ulcerans* species [71]. However, based on the LightCycler melting curve analysis using fluorescence resonance energy transfer (FRET) probes, a new group of feline *C. diphtheriae* strains—whose *tox* gene sequence is more similar to *C. ulcerans tox* than to the *tox* of human *C. diphtheriae* strains—may be misclassified as *C. ulcerans* [57,71]. Importantly, due to recent changes in the taxonomy of *C. diphtheriae*, these atypical, feline-derived strains have been proposed to be reclassified into a novel species, *Corynebacterium rouxii* [78]. To overcome the problems related to *tox* gene heterogeneity, Mancini et al. [79] developed a diagnostic algorithm combining two different real-time PCR assays based on the *tox* and *rpoB* gene amplifications with LightCycler melting curve analysis. The analysis of melting curve profiles of the *rpoB* amplicons (the gene encoding the β subunit of RNA polymerase) enabled species differentiation between *C. diphtheriae* and *C. ulcerans*, while the analysis of the *tox* gene effectively distinguished the *tox* from *C. diphtheriae* and *C. ulcerans*/*C. pseudotuberculosis* species [79]. However, as the authors noted, potential misclassification cannot be ruled out, as shown by the unexpected results of melting temperature obtained for single strains of *C. ulcerans* and *C. pseudotuberculosis*.

In addition to the above, multiplex PCR (mPCR) assays, based on conventional or real-time systems, have been designed to combine *tox* gene detection with identification of corynebacterial species [79,80,81,82,83,84]. The species-specific conventional mPCR proposed by Pimenta et al. [80] combined the detection of *toxA* and *toxB* with *C. diphtheriae* identification using primers targeting *dtxR*, the product of which acts as a global regulator of metabolism in both toxigenic and non-toxigenic strains, including the regulation of *tox* expression [80]. Another conventional mPCR based on the amplification of the *tox* and *dtxR* genes was described by Sunarno et al. [84], which, in addition to *tox* detection and differentiation of three potentially toxigenic corynebacterial species (*C. diphtheriae*, *C. ulcerans*, and *C. pseudotuberculosis*), was also used to predict some NTTB type strains (deletions at position 25 or 55 of the *tox* gene) [84]. The conventional pentaplex PCR assay proposed by Torrez et al. [81], in addition to *tox*-specific primers (a region between fragments A and B), contains primers for detection of *C. diphtheriae*, *C. ulcerans*, and *C. pseudotuberculosis*. These primers correspond to the following genes: *rpoB* for *Corynebacterium* spp. identification, 16S rRNA sequence specific for *C. ulcerans* and *C. pseudotuberculosis*—*pld* a *C. pseudotuberculosis*-specific sequence (gene encoding PLD exotoxin), and the *dtxR* gene of *C. diphtheriae*. De Zoysa et al. [83] developed and validated a quadruplex real-time PCR targeting fragment A of *tox*-carrying corynebacteria, a *rpoB* fragment specific for *C. diphtheriae*, and a second *rpoB* fragment specific for *C. ulcerans*/*C. pseudotuberculosis* [22,83]. To test PCR inhibition, an internal control in the form of green fluorescent protein (*gfp*) gene amplification after addition of control DNA to the samples was included in the assay. The assay demonstrated 100% diagnostic sensitivity, high specificity (98–100%), and 100% concordant results with those of phenotypic tests (excluding NTTB strains) and the conventional PCR of Pallen et al. [64]. The method correctly identified *C. ulcerans* strains that had previously yielded false-negative results in the Mothershed et al. test [67]. The limit of detection was lower than 1 copy per reaction for all three targets [22,83]. The results were available 3–4 h after receiving the isolate, significantly reducing the time to report a result compared to conventional PCR [83]. This test has been used in Public Health England since 2014 and is currently recommended by the WHO for identification of potentially toxigenic corynebacteria. Since the use of *gfp* as an internal control did not provide information on the efficiency of bacterial DNA extraction, this method was recently updated by replacing the *gfp* control with amplification of a 16S rRNA universal fragment [82]. This modified pentaplex real-time PCR has been subsequently validated, and its usefulness was confirmed in studies on both bacterial strains and clinical samples—on both pure DNA preparations and boiling preparations [82]. Other assays using real-time multiplex PCR have also been described for *tox* gene identification and discrimination of potentially toxigenic corynebacteria using a set of primers and probes corresponding to the *tox* and *rpoB* genes [61,85] or to the *tox* and *dtxR* genes [86].

### 5.2. Loop-Mediated Isothermal Amplification

Another molecular technique that has been recently used to detect *C. diphtheriae* and to differentiate between toxigenic and non-toxigenic strains is loop-mediated isothermal amplification (LAMP) [87]. In general, the LAMP method is a highly specific amplification reaction using strand-displacing DNA polymerase, 4–6 primers recognising 6–8 distinct regions of target DNA, which generate concatemeric products under constant temperature conditions [88]. Zasada et al. designed two LAMP assays for the detection of *tox* and *dtxR* genes [87]. The developed assays were highly specific and sensitive (100% specificity and 100% sensitivity compared to conventional PCR and Elek test), with a low detection limit estimated at 2.84 pg of DNA per reaction when the amplification was carried out for 60 min. The sensitivity of the assays decreased about 10-fold for every 10 min reduction in the reaction time and 2.84 ng of the template was the detection limit when the reaction incubation time was 30 min. The proposed tests, in addition to being simple and quick to perform, were cost-effective—they do not require a very precise heating device, as the tests were effective at temperatures from 62 to 70 °C, and their results were successfully visualised by a colour change in the reaction mixture. The authors proposed the application of various colorimetric indicators, such as hydroxynaphthol blue (HNB), QuantiFluor and calcein, as well as immunochromatographic strips when the labelled primers were used for the reaction. Colorimetric indicators added to the reaction mixture before incubation enabled a faster detection of the positive reaction in the real-time mode [87]. The proposed LAMP assay has the potential for Point-of-Care Testing (POCT) diagnosis of diphtheria and other *C. diphtheriae* infections, although this would require prior testing of their performance directly on clinical specimens.

## 6. Biosensors

Biosensors are a new technology that has been developed for diagnostic purposes in recent years. Biosensors are specific analytical probes consisting of a biological part that functions as a sensor and a transducer that converts biological changes into a signal that is proportional to the concentration of the detected agent [89]. There are two main groups of biosensors: (i) optical biosensors, that often use surface plasmon resonance (SPR), colorimetry and fluorescence; and (ii) electrochemical biosensors that are usually based on impedance, amperometry and voltammetry [90]. According to the literature, there are only a few biosensors developed for detecting DT or the DT gene. The function of biosensors for DT detection is similar to the technology used in ELISA tests, whereas the biosensors for *tox* gene detection are based on the phenomenon of specific hybridisation of DNA strands. Moreno-Bondi et al. [91] designed a biosensor consisting of a set of specific IgG antibodies that were immobilised on borosilicate microscope slides by the interaction of lysine residues of the antigens with the N-hydroxysuccinimidyl ester terminus. This biosensor allows the analysis of patients^’^ serum to detect the DT, tetanus toxin, staphylococcal enterotoxin B and hepatitis B [91]. Detection was carried out using Cy5-labelled goat anti-human IgG secondary antibodies and measuring the fluorescence level. The tests showed detection limits in the range of 0.2–3 μg of specific IgG per mL, with an absolute optical detection limit of approximately 100 fg [91]. However, the authors of the cited article indicated that a significant problem of the designed biosensor is the possibility of absorbing non-specific antigens that may be present in the serum in excess of the detected antigens. Furthermore, the high protein content as well as the high viscosity of undiluted serum may also interfere with the test. Further research is necessary to improve the diagnostic potential. Another type is the optical biosensor proposed by Zeinoddini et al. [92]. A localised surface plasmon resonance (LSPR) nanobioprobe was used to design it, in which monoclonal anti-DT IgG antibody and gold nanoparticles were conjugated. In order, fluorescence spectroscopy was used to detect territorial structural alterations in the antibody in the presence of DT [92]. The authors of the article indicate that the sensitivity of this method is about 10 ng/mL, and the time necessary to perform the test is about 1–2 h [92]. Therefore, the LSPR biosensor has high potential to be used for rapid DT detection compared to traditional methods. However, it seems that electrochemical biosensors would be the most optimal due to their simplicity of use, low measurement costs and desirable miniature size. Ziółkowski et al. [93] present an in-depth analysis, which resulted in an electrochemical biosensor with anti-DT IgG antibodies, for the immobilisation of which an intermediate cysteamine layer and a crosslinker—glutaraldehyde—were used. The antibody-based receptor layer was tethered to gold nanospheres, and BSA acted as a blocking agent to reduce non-specific adsorption [93]. The signal change against DT was calculated on the basis of reduction peak currents and charge transfer resistance, derived from square–wave voltammetry and impedance spectroscopy. The linear dependence of redox current change against DT was recorded within 10^−4^ to 10^−1^ Lf/mL, where the Lf is defined as the antigen-content-forming 1:1 ratio against 1 unit of antitoxin [93]. Even better specificity and detection level were obtained using an electrochemical biosensor in which the gold electrode surface was modified by co-adsorption of HS-L-Biotin and diethyldithiocarbamate [94]. This special layer was characterised by reduced nonspecific protein adsorption and high conductivity. Anti-DT IgG antibodies were immobilised by a biotin-streptavidin reaction. The mechanism of detection based on the differential analysis of square wave voltammetry (SWV) allowed the authors to obtain a lower detection limit at the level of 5 ‧ 10^−6^ μg/mL [94]. A very interesting concept was proposed by Ameku et al. [95]. An electrochemical immunosensor was developed in which a dipeptide consisting of two epitopes of DT was conjugated to the surface of a pencil-lead electrode integrated into a portable electrode holder. This system was able to capture anti-DT IgG antibodies for measurement by an indirect immunoassay using secondary antibodies conjugated with alkaline phosphatase, which, in turn, enzymatically generates hydroquinone, a redox molecule measurable by SWV. Under the optimised working conditions, a limit of detection of 5 ‧ 10^−6^ IU/mL was achieved [95]. For detection of the *tox* gene, Marchlewicz et al. [96] proposed an electrochemical genosensor (DNA biosensor) with the potential to be used in a portable device. The detection is based on the combination of asymmetric PCR (aPCR) and a specific stem-loop structure DNA probe immobilised on the electrode surface. The probe hybridises to the aPCR product, resulting in the changes in the electrochemical signal. The detection limit is 20.8 nM of the target (*tox* gene fragment) in 5 min or 0.5 nM in 30 min.

## 7. Variations in the Nucleotide and Amino Acid Sequences of DT

Changes in the nucleotide sequences of the *tox* gene and in the amino acid sequences of the toxin may strongly influence the sensitivity of NAAT and immunological-based diagnostics, respectively. As has been mentioned in the beginning of this paper, the DT gene (*tox*) occurs in some corynephages (mainly β) and by lysogenic conversion, and it may be integrated with the chromosome of corynebacteria, primarily *Corynebacterium diphtheriae* but also *C. pseudotuberculosis, C. ulcerans* and others [6,97]. Comparative genomic studies of 12 *C. diphtheriae* clinical strains in relation to references *C. diphtheriae* NCTC 13129 showed great diversity of the sequenced *tox*-positive corynephage integrated with the bacterial genomes but not along its entire length. The right side of the prophage genomes is characterised by the greatest nucleotide sequence similarity. This region harbours the *tox* gene and is specified by a decreased GC content [98]; hence, there is practically no variability observed within the DT gene [99]. Even though *tox* is extrinsic, it is regulated by a chromosomally encoded protein DtxR (DT repressor), which is a transcriptional regulator that uses Fe^2+^ as a corepressor [100].

Research on identifying the sequence of the DT and DtxR structural genes began in the early 1980s [101,102] and 1990s [7], respectively. This paved the way to analysing the evolution of these genes among isolates originating from different places and at different times. PCR-single-strand conformation polymorphism analysis (PCR-SSCP) of 72 *C. diphtheriae* clinical strains isolated in Russia and Ukraine showed heterogeneity of the *tox* and *dtxR* genes [103]. Further analysis by sequencing revealed that in the case of the *tox* gene, there were three different point mutations: one in the A subunit and three in the B subunit, but all four were silent. Significantly more heterogeneity was detected in the *dtxR* gene, namely 35 point mutations were detected, 9 of which resulted in amino acid substitutions in the carboxyl-terminal half of DtxR and 26 of which were silent [66].

Much more recent research performed in Indonesia confirms these observations [99]. Among 89 throat swabs from patients with a clinical diagnosis of diphtheria, 10 toxin-producing *C. diphtheriae* isolates were detected, for which the *toxB* sequence was analysed. Nine isolates showed an SNP mutation (G30A), but the mutation did not change the amino acid sequence of the toxin. In consequence, the authors point to other factors that could have reduced the efficacy of the diphtheria vaccine used in Indonesia, for example, mutations in the toxin promoter/operator region (*tox* PO) [99]. The results of the research by Kolodkina et al. showed that the increase in the level of DT production is determined by the mutation located in the 9-bp palindrome, which overlaps the –10 sequence of the promoter and the operator region [104].

Despite the emphasis on the fact that DT produced by *C. diphtheriae* is highly conserved at the amino acid level [66,99,105], it is worth noting that there are few reports showing heterogeneity of *tox* sequences between and within *Corynebacterium* species [73,106]. Comparative analysis of *tox* sequences of the *C. diphtheriae* and three *C. ulcerans* strains resulted in remarkable differences at the nucleotide but also at the amino acid level, mainly in the B fragment of DT. Inter- and intra-species variability has been proven and it has also been shown that DT amino acid sequences from a *C. ulcerans* isolate associated with classical diphtheria are less well conserved than those from *C. diphtheriae* [106]. It is extremely important to take this fact into account when diagnosing diphtheria and carefully design primers and probes for detecting the *C. ulcerans tox* gene using NAATs [74].

## 8. Conclusions

In the history of diphtheria diagnostics, three turning points might be identified: (i) the demonstration that DT is a major virulence factor of *C. diphtheriae* and development of the toxigenicity test using an animal model in 1888; (ii) the development of agar immunodiffusion assay for the detection of DT production in 1948, commonly called the Elek test; and (iii) the development of PCR in 1991, which enables the detection of the *tox* gene. Many other various methods for the detection of DT and identification of toxigenic isolates have been proposed but the sensitivity, repeatability and reproducibility of most of them have not been confirmed on a sufficiently large group of bacterial isolates or clinical samples (Table 1).

Regulations in force in the European Union (EU) require implementation of the following three principles concerning the use of animals for laboratory tests: replacement, reduction and refinement (the 3Rs) [107]. Therefore, the toxigenicity test using an animal model is not used any more in the EU for diagnostic purposes. Despite enormous progress in science and diagnostics of infectious diseases, after almost 80 years, the Elek test is still the basic recommended diagnostic test, enabling the detection of the main pathogenicity factor of *C. diphtheriae*, which is a highly potent toxin. Although the diphtheria vaccine has been known for many decades and has been broadly used in many countries, the disease has not been eradicated. In the 21st century, diphtheria occurs not only as single cases but also as epidemics in some regions of the world. Moreover, new *Corynebacterium* species with the potential to produce DT were described [5,6]. Therefore, a rapid, easy to interpret and modern method for the detection of DT-producing isolates is needed, especially considering that the availability of diphtheria antitoxin—which is a main cure and a necessary reagent for current diagnostics methods—is extremally limited. The very promising tools are ICS and LFIA for toxin detection, and isothermal amplification methods, such as LAMP, for the detection of *tox* gene. Also, biosensors represent a promising technology for the rapid DT detection in clinical samples, but they still face challenges that must be addressed to maximise the sensitivity and selectivity of the test and to eliminate cross-reactivity.

According to the definition for the purposes of epidemiological surveillance, a confirmed diagnosis of diphtheria requires meeting both clinical and laboratory criteria. The laboratory criterion includes the isolation of toxin-producing corynebacteria from clinical material. Local public health laboratories deal with initial diagnostics consisting of isolation and molecular identification. Due to the fact that PCR only allows for the detection of the gene encoding DT but does not provide information on the production of DT, it is necessary to perform the Elek test in all *tox*-positive isolates to confirm whether the strain is toxigenic. Diagnostic laboratories are recommended to submit *tox*-positive isolates to the National Reference Laboratory to confirm the production DT with the Elek test. Currently, the role of Reference Laboratories is crucial for diphtheria diagnostics because these are the only laboratories with access to diphtheria antitoxin necessary for the Elek test and where staff have the necessary experience in the conduction and result interpretation of the Elek test. However, the necessity to transport the sample to the Reference Laboratory requires additional time, which results in a delay in obtaining information about the toxigenicity status of the isolate, introducing an appropriate therapy to the patient, and taking actions to prevent the spread of the infection. The challenges in the development of new test for DT detection include, among others, the following: (i) the test should be independent of the availability of diphtheria antitoxin; (ii) it should be easy-to-perform and the results should be easy-to-interpret so that local laboratories would be able to carry out the test even without any experience in diphtheria diagnostics; (iii) it should be rapid; (iv) it might be implemented as a point-of-care test, especially in cases when an outbreak is suspected; and (v) it should be easy to store and maintain activity for long time because diphtheria cases occur very rarely in some countries.

## Figures and Tables

**Table 1:** Detection methods for diphtheria toxin.

Technique	Method	LOD [ng/mL]	Time to Obtain the Result ^f^	Experience of Laboratory Staff	Comments
In vivo ^a^	Animal model	ND	48–120 h	very high	traditional “gold standard”; animal facility required; not recommended due to the 3R rule
Cell lines ^a^	In vitro tissue culture cytotoxicity	0.01–0.1	24–120 h	very high	specialized laboratory facilities required; mainly used for measuring serum antitoxin levels
Immunological ^a^	Elek test	ND ^d^	24–48 h 16–24 h ^g^	very high	basic recommended diagnostic test; prone to misinterpretation; technically demanding
	Counterimmunoelectrophoresis (CIE)	ND	16–24 h	average	evaluated on a small number of isolates
	Agglutination methods	0.2–5	12 h	average	evaluated on a small number of isolates
	Enzyme-linked immunosorbent assay (ELISA)	<1	22 h	high	no commercially available kits; recommended by WHO for detection and quantification of anti-DT antibodies
	Enzyme immunoassay (EIA)	0.1	3 h	average	easy to interpret
	Immunoblotting	5–10	11–24 h	average	not recommended for routine diagnostic use
	Immunochromatographic strip test (ICS)	0.5	3.5–6.5 h	low	easy to apply and interpret
Molecular ^b^	Polymerase Chain Reaction (PCR)	2.5 ^e^	4–5 h ^h^	average	allows to detect NTTB strains; must be confirmed by the Elek test; specialized laboratory facilities required
	Real-time PCR	<0.017 ^e^	3–4 h ^h^	high	
	Loop-mediated isothermal amplification (LAMP)	2.84 ^e^	1 h ^h^	average	not require a very precise heating device; potential Point-of-Care Testing; allows to detect NTTB strains; must be confirmed by the Elek test
Optical/electrochemical ^c^	Biosensors	0.005–10	1–2 h	low	potential Point-of-Care Testing, allows to detect NTTB strains (genosensors); genosensors’ results must be confirmed by the Elek test

LOD—limit of detection; ND—not determined; ^a^—DT detection; ^b^—*tox* gene detection; ^c^—depends of type, DT or *tox* gene detection; ^d^—modified Elek test may detect toxin even in low-toxigenic strains; ^e^—pg of genomic DNA/reaction; ^f^—time counted from the moment of obtaining a pure culture; ^g^—modifications; ^h^—without DNA isolation.
